# Compensatory relationships determine the impact of TGF-β on the humoral immune response to hepatitis B surface antigen

**DOI:** 10.1093/jimmun/vkag129

**Published:** 2026-06-17

**Authors:** Jesse L Cimino, Safiehkhatoon Moshkani, Jacob T Bailey, Catherine Rexhouse, Mitchell J Waldran, Michael D Robek

**Affiliations:** Department of Immunology & Microbial Disease, Albany Medical College, Albany, NY, United States; Department of Immunology & Microbial Disease, Albany Medical College, Albany, NY, United States; Department of Immunology & Microbial Disease, Albany Medical College, Albany, NY, United States; Department of Immunology & Microbial Disease, Albany Medical College, Albany, NY, United States; Department of Immunology & Microbial Disease, Albany Medical College, Albany, NY, United States; Department of Immunology & Microbial Disease, Albany Medical College, Albany, NY, United States

**Keywords:** antibodies, B cells, cytokines, viral

## Abstract

Despite an effective vaccine against the hepatitis B virus (HBV), there are about 250 million people living with chronic HBV (CHB) worldwide and one million deaths annually. Most children and about 5% of adults exposed to HBV will fail to clear the virus, developing a lifelong infection. Hepatitis B surface antigen (HBsAg)-specific antibody (HBsAb) is protective in uninfected individuals and is considered a component of a functional cure. Immune factors that play important roles in regulating inflammation, such as TGF-β, IL-10, and regulatory T cells (Tregs), may also contribute to CHB pathogenesis. However, the early regulatory factors that promote HBsAg seroconversion are not well understood. To address this, we utilized adeno-associated virus (AAV)-mediated delivery of HBV (AAV-HBV) to mice. In this model, C57BL/6 mice fail to develop effective HBsAb responses, while BALB/c mice more efficiently seroconvert HBsAg. While inhibiting TGF-β, IL-10, or Tregs did not impact serum HBsAg levels in C57BL/6 mice, TGF-β depletion in BALB/c mice ablated the humoral response to HBsAg. Neutralizing IL-10, blocking CTLA-4, or depleting Tregs alone did not affect the HBsAb response in BALB/c mice. However, Treg depletion in the absence of TGF-β restored HBsAg clearance in an IL-10- and CTLA-4-independent manner. These findings highlight the immune balance regulated by TGF-β in the early adaptive response to an HBV antigen, as well as context-dependent compensatory interactions that may directly or indirectly impact antigen-specific humoral immunity.

## Introduction

Potent antigen-specific antibody responses are essential for preventing viral infection and limiting spread, whereas effective control and clearance of established infections typically require durable virus-specific T cell immunity. The early and robust activation of B and T lymphocytes, facilitated by antigen-presenting cell (APC) costimulation, culminates in the formation of germinal centers (GCs) and the production of mature IgG class-switched antibodies.^1–3^ Immune regulatory factors, such as regulatory T cells (Tregs) and the cytokines transforming growth factor β (TGF-β) and interleukin-10 (IL-10), have been implicated in playing various, context-dependent roles in adaptive immune responses to viral infections.^4–11^

During infection by the liver-tropic hepatitis B virus (HBV), the absence of a potent HBV surface antigen (HBsAg)-specific antibody response is associated with a lifelong chronic infection that often results in liver cirrhosis or hepatocellular carcinoma.^12^^,^^13^ Seroconversion occurs in patients who control HBV, in which circulating HBsAg and HBV virions diminish, accompanied by the appearance of HBsAg-specific IgG antibodies (HBsAb). These antibodies prevent further viral spread to uninfected hepatocytes in the liver by neutralizing virion binding to the entry receptor.^13^ This HBsAg seroconversion is considered part of a “functional cure,” and HBsAb is found in most patients who have cleared HBV or received HBV immunization.^14^^,^^15^ Some individuals fail to generate an adequate HBsAb response to the HBV vaccine,^16^ further warranting a better understanding of the immune factors that drive this response. However, due to the hepatic nature of HBV infection, the challenge of acquiring patient samples during early acute infection, and the limitations of existing animal models, there is a knowledge gap regarding the critical immune regulatory factors that promote seroconversion. Thus, identifying these factors and the underlying mechanisms that promote seroconversion and viral antigen clearance is of high importance.

The immune response to HBV is challenging to evaluate experimentally due to a limited natural viral tropism that includes humans and some primates, but not rodents.^17^^,^^18^ Adeno-associated virus (AAV) delivery of the HBV genome (AAV-HBV) into mice initiates viral replication, effectively establishing continued HBV replication in infected hepatocytes, including the secretion of virions and viral antigens into the periphery.^19–21^ This immunocompetent model mirrors peripheral B and T cell tolerance and hepatic immune priming observed in human infection, but is limited in part by the inability of newly secreted HBV virions to spread to uninfected hepatocytes.^20^ AAV-HBV-transduced C57BL/6 mice exhibit sustained T and B cell tolerance towards HBsAg that mimics chronic infection in humans, while BALB/c mice display reduced B cell tolerance with the ability to clear peripheral HBsAg.^21–24^ Yet neither strain spontaneously eliminates HBV replication without additional immune intervention, as both lack the polyfunctional CD8^+^ T cell response required to eliminate HBV-infected hepatocytes.^21–24^

Tregs and the immunoregulatory cytokines TGF-β and IL-10 have been associated with CHB disease, where their elevated expression and suppressive capabilities may contribute to immune suppression and viral persistence.^7–9^^,^^25^ Furthermore, these factors can not only impact the critical T and B lymphocytes that are responsible for adaptive immune responses, but they also function to preserve immune balance during homeostasis and inflammatory events.^4–6^ Tregs are primarily governed by TGF-β, and can modulate adaptive immunity through multiple mechanisms including IL-10, CTLA-4-mediated inhibition, PD-1 axis engagement, IL-2 sequestration, and regulation of immune cell metabolism.^26^ However, despite being recognized as pleotropic regulators of immune balance, the specific roles of these factors in shaping the early antiviral immune response to HBV, including the development of humoral immunity against HBsAg, are poorly defined.

To address this, we used the AAV-HBV mouse model to examine how TGF-β influences the initiation of HBV-specific antibody responses. We found that TGF-β contributes to early humoral priming against HBsAg and that, in its absence, other regulatory mechanisms directly or indirectly impact the HBsAb response. These results highlight an underappreciated role for TGF-β and Treg-mediated signaling in coordinating the early antibody response to HBV, with implications for strategies to promote seroconversion and achieve functional cure.

## Materials and methods

### Mice

Seven-week-old male C57BL/6 (strain #000664) and BALB/c (strain #000651) mice were purchased from The Jackson Laboratory and housed in the Animal Resource Facility at Albany Medical College. All experiments followed protocols approved by the Albany Medical College Institutional Animal Care and Use Committee.

### AAV-HBV transduction

Mice were given AAV serotype 8 encoding a 1.2-mer HBV genome (Genotype D; SignaGen) intravenously via retro-orbital injection. AAV-HBV was administered at 2–4 × 10^10^ genome copies per mouse.

### Antibody administration

Unless otherwise noted in figure legends, antibody was typically administered intraperitoneally twice weekly at a dose of 250 µg. Injections began five days before AAV-HBV transduction and continued for a total of 8 or 10 doses per mouse. Mouse α-TGF-β (clone 1D11.16.8), α-CD25 (PC-61.5.3), α-IL-10 (JES5-2A5), α-IL-10R (1B1.3A), α-CTLA-4 (9H10), α-PD-1 (RMP1-14), α-IL-21R (4A9), mouse IgG1 isotype (MOPC-21), and rat IgG1 isotype (TNP6A7) antibodies were purchased from BioXCell. All non-isotype antibodies were utilized to either deplete, block, or neutralize the immune target.

### Immunization

Mice were immunized with 2 × 10^6^ PFU of vesicular stomatitis virus expressing middle HBV surface antigen (VSV-MHBs).^27^ Immunizations were performed either intramuscularly or intranasally, as indicated. Mock immunization was performed using 1× PBS.

### ELISA

Serum HBsAg, HBeAg, HBeAb, HBcAb, and HBsAb were measured by ELISA (International Immunodiagnostics). Recombinant HBsAg (subtype ayw) and HBeAg proteins (Biosynth) were utilized to create a standard curve for quantification. Serum TGF-β was also measured by ELISA (R&D Systems Human/Mouse/Rat/Porcine/Canine TGF-β1 Quantikine ELISA). For total serum TGF-β measurement, acid treatment steps that separate TGF-β from latent peptide were included, while for active serum TGF-β assessment, these steps were excluded. Serum IL-2 was measured using a Mouse IL-2 Quantikine ELISA kit (R&D Systems), and serum IgG was measured using a mouse total IgG ELISA kit (Thermo Fisher).

### ELISPOT

IgG ELISPOT was performed according to the manufacturer’s protocol (Mouse IgG/IgM double-color ELISPOT assay, Cellular Technology Limited). Briefly, 96-well plates were coated overnight at 4 °C with 100 µl of 10 µg/mL recombinant HBsAg (Biosynth), bovine serum albumin (BSA), or no antigen. Two hundred thousand mouse splenocytes were added per well to the washed plate, and the plates were incubated overnight at 37 °C. After ELISPOT detection steps, IgG antibody spots were quantified automatically using an ImmunoSpot counter (Cellular Technology Limited). IFN-γ ELISPOT was performed according to the manufacturer’s protocol (Mouse IFN-γ ELISPOT assay, BD Biosciences). Briefly, plates were coated overnight with α-IFN-γ antibody, and the next day, 2 × 10^5^ cells were added per well. Cells were stimulated overnight with 10 µg/ml HBV peptides. Quantification of IFN-γ spot-forming cells was performed as indicated above. Peptides used for restimulation were HBs 191 [IPQSLDSWWTSL], HBs 353 [VWLSVIWM], HBs 364 [WGPSLYSIL], HBs 371 [ILSPFLPL], Core 87 [SYVNTNMGL], and Core 93 [MGLKFRQL]. For both assays, spot counts from background wells (BSA or no antigen/peptide) were subtracted.

### Flow cytometry

Spleen or perfused liver were collected from euthanized mice, and immune cells were isolated and processed for flow cytometry. Spleens were collected into RPMI media containing 1% FBS and homogenized by pressing through a 70 µm strainer. RBC lysis was performed using ACK lysis buffer, and samples were resuspended in RPMI containing 1% FBS before staining. Liver was collected in Hank’s Balanced Salt Solution (HBSS; Sigma), and minced liver was digested in 0.05% collagenase type IV before being passed through a 70 µm filter. Leukocytes were isolated by resuspending homogenates in 40% Percoll in PBS followed by centrifugation (560 × g, 15 min) before RBC lysis and were resuspended in 5% FBS in HBSS. Cells were stained for 20 min on ice with antibodies purchased from BioLegend: α-GL7 (APC, GL7), α-CD138 (PE, 281-2), α-CD3 (FITC, 17A2), α-CD4 (APCCy7 and FITC, Gk1.5), α-CD40L (PE, SA047C3), α-CXCR5 (APC, L138D7), α-PD-1 (BV421, 29F.1A12), α-CTLA-4 (BV421, 4C10-4B9), α-ICOS (PECy7, C398.4A), and α-IL-21R (PE; 4A9); or BD Biosciences: α-CD19 (APCH7, 1D3); or Thermo Fisher: α-CD25 (APC, PC61.5), α-FoxP3 (PE, FJK-16s), α-CD4 (FITC, RM4-5), and α-CD19 (PECy5, 1D3). Cells were then washed and fixed via 10-min incubation in 4% paraformaldehyde in PBS on ice before being pelleted by centrifugation and resuspended in FACS buffer. For FoxP3, cells were fixed, permeabilized, and stained using a Mouse Treg Staining Kit (Thermo Fisher) according to the manufacturer’s protocol.

Flow cytometry gating for every analysis began by gating on singlets (FSC-H vs FSC-W; FSC-H vs FSC-A) before gating on lymphocytes (SSC-A vs FSC-A). Cell populations of interest were then gated as detailed in the corresponding figure legends. For compensation, unstained controls and single-color controls were used for each individual target. Fluorescence minus one controls were incorporated as necessary. All samples were processed using a BD FACSymphony A3 flow cytometer, and data were analyzed using FloJo software version 10.

### Gene expression (RT-qPCR)

Liver and spleen samples were snap-frozen and stored at −80°C before being homogenized in RLT buffer with 2-mercaptoethanol. RNA was then extracted utilizing an RNeasy mini kit (Qiagen). A High-Capacity cDNA Reverse Transcription Kit (Thermo Fisher) was used to generate cDNA, corresponding with the manufacturer’s protocols. TaqMan Fast Advanced Master Mix (Thermo Fisher) was used for the polymerase chain reaction (PCR). The reactions were performed using a QuantStudio 6 real-time PCR system (Thermo Fisher) and analyzed by QuantStudio Design and Analysis software v2. The primer sequences utilized for HBV RNA detection were HBV probe 5′-CCT CTT CAT CCT GCT GCT ATG CCT CAT C-3′, antisense 5′-GAC AAA CGG GCA ACA TAC CTT-3′, sense 5′- GTG TCT GCG GCG TTT TAT CA-3′.^28^ Taqman assays (Thermo Fisher) included IFN-γ (Mm01168134_m1), CD4 (Mm00442754_m1), CD8 (Mm01182107_g1), CD40L (Mm00441911_m1), CD40 (Mm00441891_m1), IL-2Rα (Mm01340213_m1), IL-12A (Mm00434169_m1), AICDA (Mm01184115_m1), ICOSL (Mm00497237_m1), H2-AB1 (Mm00439216_m1), CD39 (Mm00515447_m1), IDO1 (Mm00492586_m1), CD86 (Mm00444540_m1), CTLA-4 (Mm00486849_m1), GZMB (Mm00442837_m1), Ki67 (Mm01278617_m1), PD-1 (Mm01285676_m1), PD-L1 (Mm03048248_m1), and RNA expression was normalized to GAPDH (Mm99999915_g1).

### HBV DNA qPCR

Serum samples were collected from mice, and HBV DNA was isolated using a High Pure Viral Nucleic Acid Kit (Roche). Briefly, 20 µl of serum was diluted in 1x PBS and then combined with proteinase K, followed by a 30-min incubation at 72 °C. Next, DNA samples were purified in spin columns before elution in RNAse-free water. HBV PCR was performed using TaqMan Fast Advanced Master Mix (Thermo Fisher), and reactions were performed with a QuantStudio 6 real-time PCR system (Thermo Fisher) before subsequent analysis using QuantStudio Design and Analysis software v2. A plasmid containing the HBV genome was utilized to generate a standard curve for quantification. Primers used for HBV detection are described in the previous section.

### VSV neutralization assay

Mouse serum samples were serially diluted and incubated with 100 PFU of VSV for 1 h. Diluted virus and serum were then added to Baby Hamster Kidney (BHK-21) cells and incubated for at least 2 d before analysis. Media was monitored for color change, and microscopy was used to confirm cytopathic effects and neutralization.

### Statistical analysis

All statistical analyses were performed using GraphPad Prism version 10.

## Results

### TGF-β promotes HBsAg clearance in BALB/c mice

**Figure 1:**
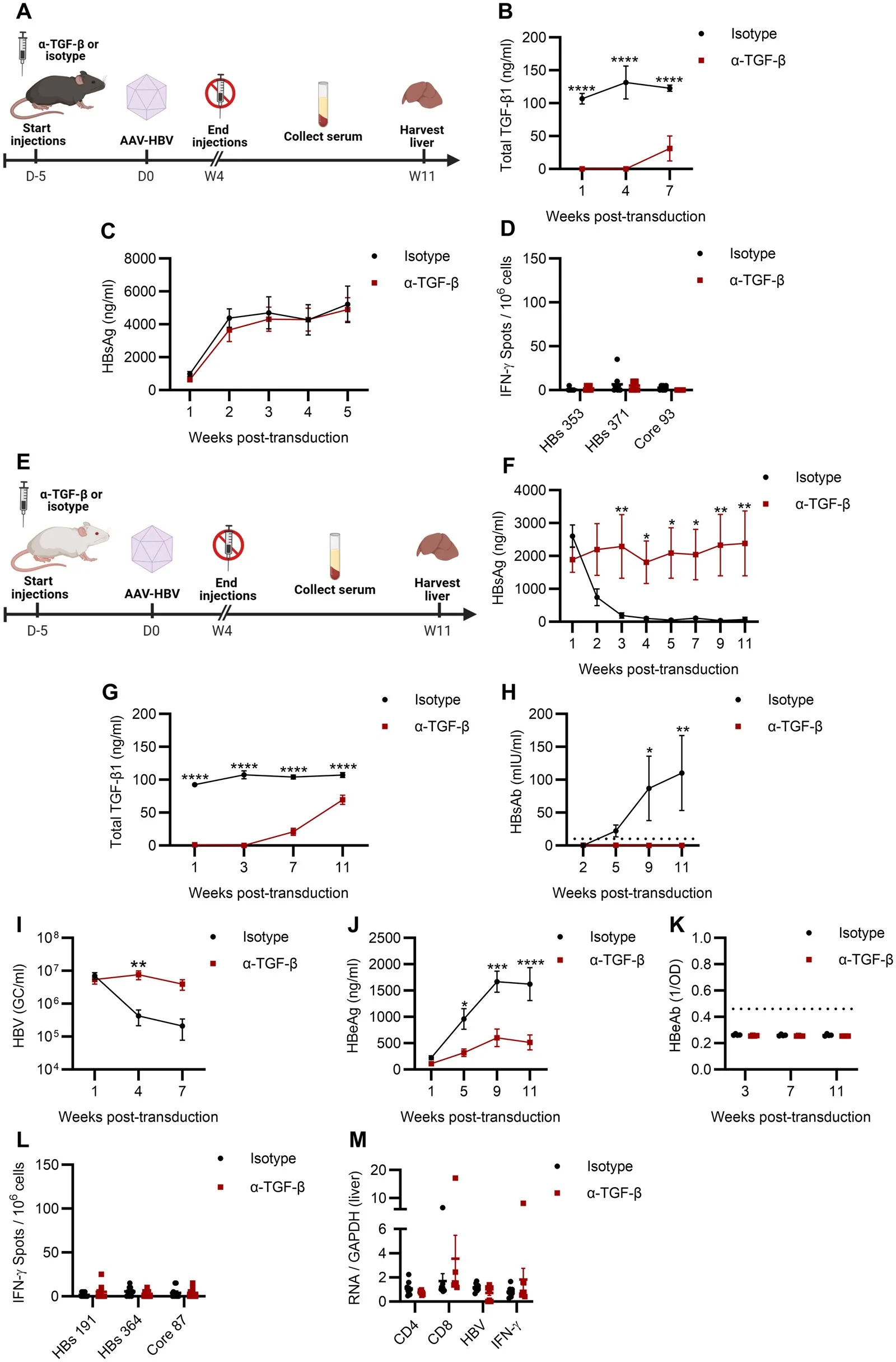
TGF-β promotes HBsAg clearance in BALB/c mice. (A–D) C57BL/6 mice or (E–M) BALB/c mice were administered α-TGF-β or isotype control antibody beginning 5 d prior to AAV-HBV transduction, continuing twice weekly for 10 total doses, and serum and liver were collected. (A, E) Experimental schema created in BioRender. Cimino, J. (2026) (A) https://BioRender.com/p2dinsu, (E) https://BioRender.com/f4vp7ce. Serum ELISAs were performed to measure (B, G) total TGF-β1, (C, F) HBsAg, (H) HBsAb, (J) HBeAg, and (K) HBeAb over time. Dotted lines represent the limit of detection. (D, L) Splenocyte T cell IFN-γ production in response to various HBV peptides was assessed via ELISPOT assay at the week 11 experimental endpoint, and background was subtracted from wells with no peptides added. (I) Serum HBV genome copies were quantified over time by qPCR. (M) RT-qPCR was performed to quantify liver RNA collected at the week 11 endpoint. The mean ± SEM is indicated; *N* = 8–10 mice per group. Each data point in (D), (K), (L), and (M) represents an individual mouse. Statistical significance was determined using two-way ANOVA with Šidák’s test for multiple comparisons. *P < 0.05, **P < 0.01, ***P < 0.001, ****P < 0.0001.

Tregs, IL-10, and TGF-β are considered critical regulatory factors that promote immune tolerance, inflammatory balance, and have well-documented relationships with chronic diseases, including HBV.^4–7^^,^^25^ In CHB infection, Tregs and immunosuppressive cytokines, such as TGF-β and IL-10, contribute to viral persistence by limiting antiviral T and B cell responses. Notably, TGF-β is frequently elevated in the liver and serum of chronically infected patients, where it is associated with impaired effector T cell function and immune tolerance.^29^^,^^30^ Therefore, we hypothesized that these factors could also influence the chronic presentation of HBV in C57BL/6 mice, including the development of persistent HBs antigenemia post-AAV-HBV transduction. TGF-β was neutralized by administration of α-TGF-β antibody before AAV-HBV transduction, and serum HBsAg levels were assessed to determine the impact on the immune response to HBV. ELISA confirmed the absence of detectable TGF-β; however, TGF-β loss did not promote HBsAg clearance or improve HBV-specific T cell responses in the spleen compared to isotype-treated control mice (Fig. 1A-D).

BALB/c mice, unlike C57BL/6 mice, efficiently generate HBs-specific antibody responses following AAV-HBV administration, which mediate the clearance of circulating HBsAg.^23^^,^^24^ However, despite this seroconversion, BALB/c mice, similar to C57BL/6 mice, do not clear HBeAg or eliminate HBV-infected hepatocytes without additional immune stimulation.^22–24^ Given the contrasting HBsAb responses between the strains, we determined whether these immune regulatory factors could have strain-specific effects on the humoral response. Neutralizing TGF-β before AAV-HBV transduction in BALB/c mice significantly impaired HBsAg clearance (Fig. 1F), and this effect persisted after detectable TGF-β returned following the cessation of antibody administration (Fig. 1G). Serum HBsAb levels were ablated in TGF-β-inhibited mice, and these mice also had elevated serum HBV DNA compared to isotype controls (Fig. 1H and I). Control mice had significantly higher serum HBeAg levels than TGF-β-depleted mice (Fig. 1J), although there was no detectable serum HBeAb in either treated or control mice (Fig. 1K). However, HBV-specific T cell responses in the spleen and HBV gene expression in the liver were unchanged at the experimental endpoint (Fig. 1L and M), suggesting that CD8^+^ T cell responses were not responsible for the loss of HBeAg. Together, these results indicate a positive role for TGF-β in promoting the antibody response to HBsAg in BALB/c mice.

### TGF-β is required for an efficient anti-HBs IgG response in AAV-HBV-transduced BALB/c mice

**Figure 2:**
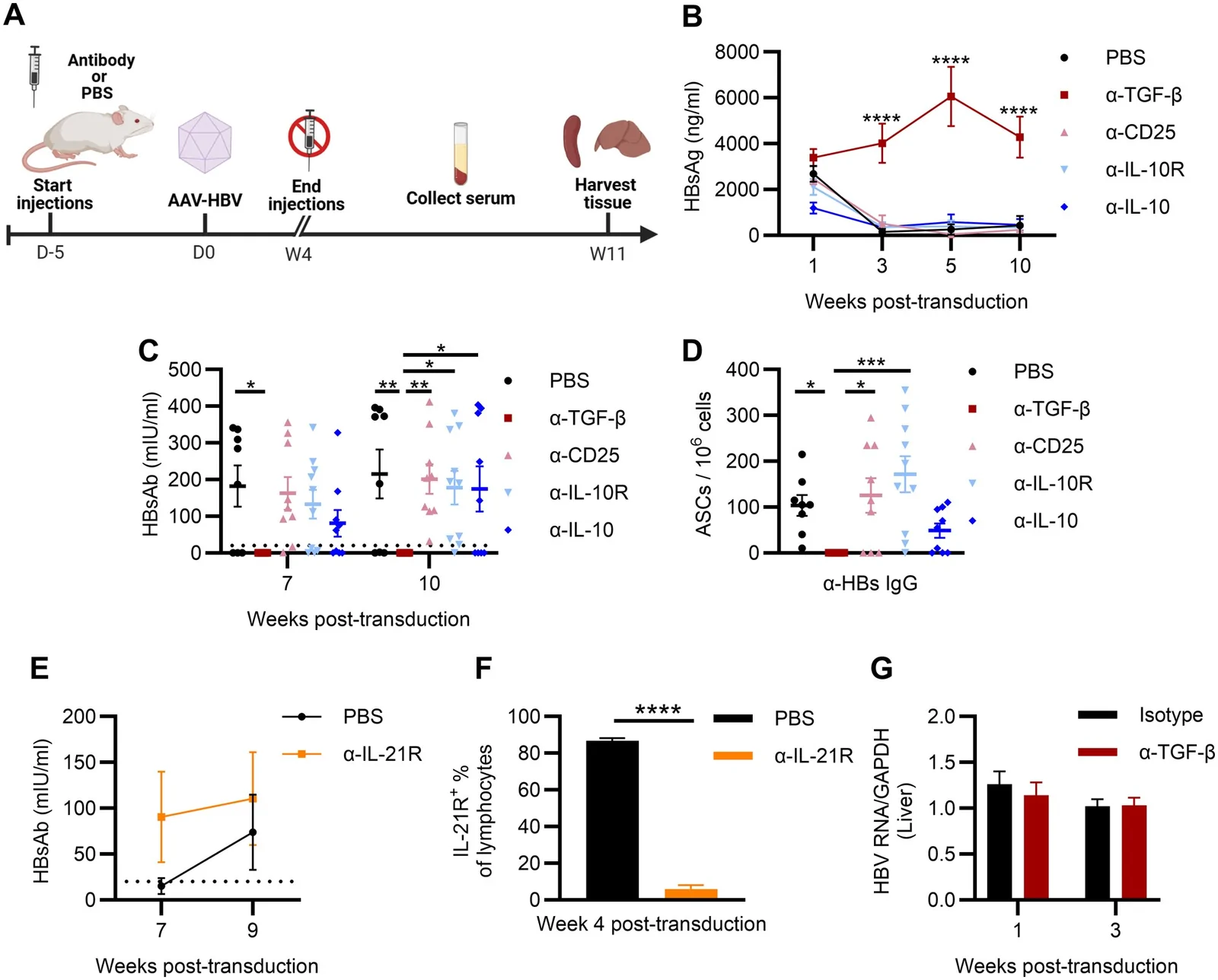
TGF-β is unique amongst other immune regulatory factors in promoting the anti-HBs IgG response. (A–F) BALB/c mice were administered α-TGF-β, α-CD25, α-IL-10, α-IL-10R, α-IL-21R, or PBS control beginning 5 d prior to AAV-HBV transduction, continuing twice weekly for 10 total doses, and serum, spleen, and liver were collected. (A) Experimental schematic created in BioRender. Cimino, J. (2026) https://BioRender.com/3wkeguf. ELISAs were performed to measure (B) serum HBsAg and (C, E) HBsAb. (D) Splenocytes were isolated at the week 11 endpoint, and HBs-specific IgG antibody-secreting cells (ASCs) were quantified by ELISPOT analysis. (F) Blood was collected from PBS or α-IL-21R-treated mice, and lymphocytes were assessed for IL-21R blockade by flow cytometry (gating on IL-21R^+^). (G) BALB/c mice were administered α-TGF-β or isotype control antibody beginning five days prior to AAV-HBV transduction, continuing twice weekly, until week 1 or week 3 post-AAV-HBV, when mice were euthanized, and the liver was harvested for RT-qPCR analysis of HBV RNA expression. The mean ± SEM is indicated; *N* = 3–10 mice per group. Each data point in (C) and (D) represents an individual mouse. Statistical significance was determined using 1-way or 2-way ANOVA with Kruskal–Wallis or Tukey’s test for multiple comparisons. *P < 0.05, **P < 0.01, ***P < 0.001, ****P < 0.0001.

The HBsAb response is known to be IgG-mediated,^31^^,^^32^ and a robust response resulting in mature HBs-specific IgG antibodies depends on activation of HBs-specific B cells and CD4^+^ T cells with the help of antigen-presenting cells (APCs).^13–15^^,^^33–35^ Previous studies have demonstrated this in the AAV-HBV model, as depleting B cells or CD4^+^ T cells, or blocking MHC II, inhibited the HBsAb response.^22^^,^^23^ Since we observed an impaired HBsAb response in the absence of TGF-β (Fig. 1H), we further investigated HBs-specific antibody production. Additionally, we assessed whether IL-10 and Tregs may impact HBsAg clearance in BALB/c mice (Fig. 2A). HBsAg clearance and serum HBsAb responses were reduced only in TGF-β-neutralized BALB/c mice, but not in those with IL-10 signaling impaired or Tregs depleted (Fig. 2B and C). Correspondingly, ELISPOT analysis of TGF-β-inhibited mice revealed a significant loss of HBs-specific IgG antibody-secreting cells (ASCs) in the spleen compared to PBS-treated mice and mice treated with α-CD25, α-IL-10R, or α-IL-10, all of which generated HBsAg-specific IgG (Fig. 2D).

IL-21 plays an important role in promoting effective IgG class-switched responses.^36^^,^^37^ Thus, to elucidate how TGF-β may regulate this, we next investigated whether IL-21 is critical for developing the HBsAb response, and found that IL-21R blockade in BALB/c mice was not sufficient to impair HBsAb production (Fig. 2E and F). We also confirmed that there was no difference in liver HBV RNA levels between TGF-β-inhibited mice and control mice post-AAV-HBV transduction (Fig. 2G). Therefore, the impaired viral clearance in the absence of TGF-β is due to the ablated HBs-specific IgG response and not a direct effect of TGF-β on HBV gene expression in infected hepatocytes. Together, these data show that TGF-β is critical to the HBs-specific IgG response and that its mechanism of action is unlikely to involve IL-21.

### TGF-β is required for the HBsAb response early post-AAV-HBV transduction

**Figure 3:**
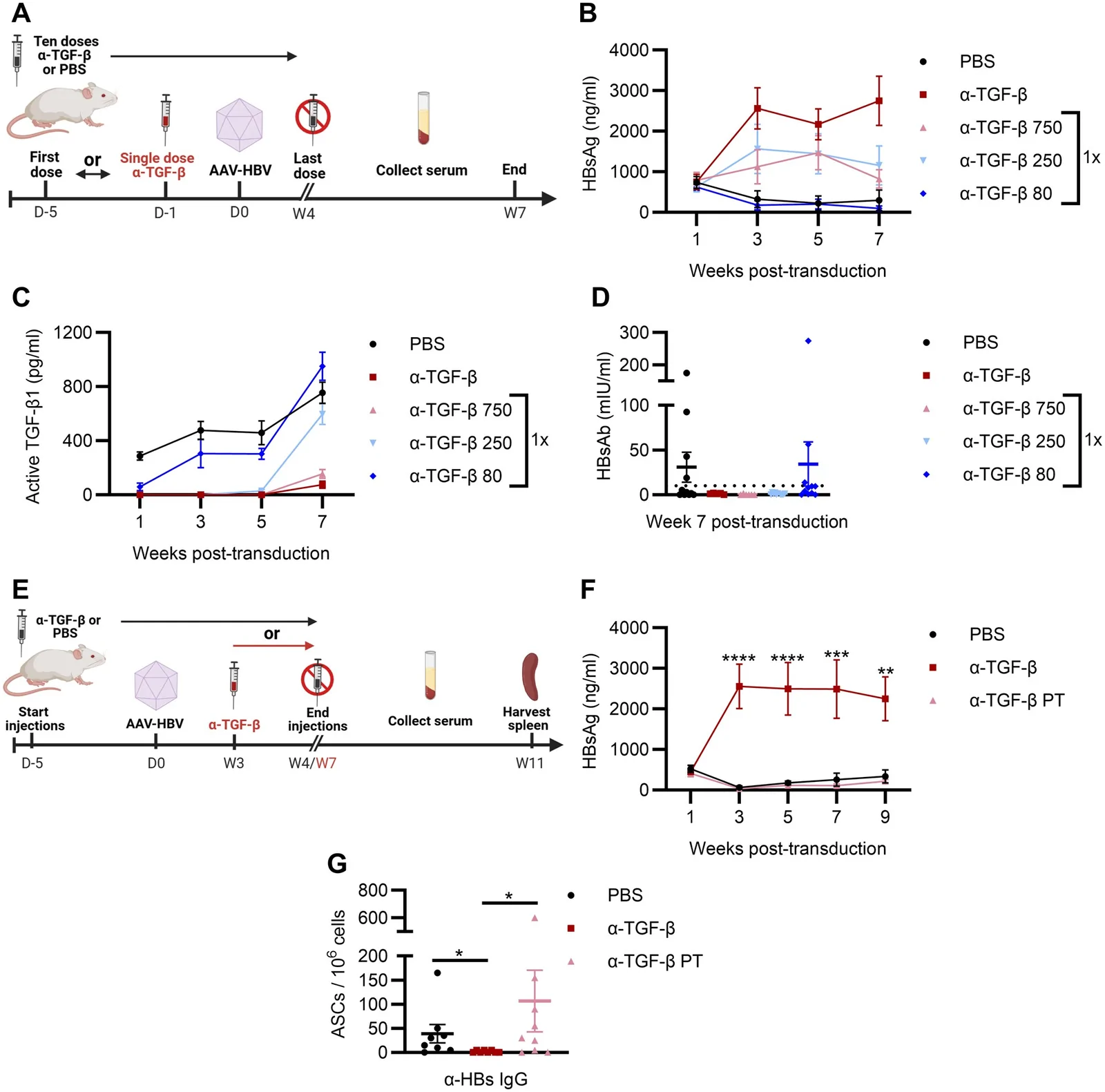
TGF-β is required for the HBsAb response early post-AAV-HBV. (A–D) BALB/c mice were administered α-TGF-β or PBS as previously described, or given a single injection of varying concentrations of α-TGF-β (750, 250, or 80 µg) one day prior to AAV-HBV, and serum was collected. (A, E) Experimental schema created in BioRender. Cimino, J. (2026) (A) https://BioRender.com/fn9q17p, (E) https://BioRender.com/onp3x51. ELISAs were performed to detect (B) HBsAg, (C) active TGF-β, and (D) HBsAb. Data shown are from two experiments with *N* = 5–6 mice per group per experiment. Dotted lines represent the limit of detection. (E–G) BALB/c mice were administered α-TGF-β or PBS beginning five days prior to AAV-HBV transduction or α-TGF-β beginning 3 wk post-AAV-HBV transduction (PT), continuing twice weekly for ten total doses, and serum and spleen were collected. (F) HBsAg over time by ELISA. (G) Splenocytes were isolated at the week 9 endpoint for quantification of HBs-specific IgG ASCs by ELISPOT. The mean ± SEM is indicated; *N* = 8–9 mice per group. Each data point in (D) and (G) represents an individual mouse. Statistical significance was determined using 1-way or 2-way ANOVA with Kruskal–Wallis or Tukey’s test for multiple comparisons. *P < 0.05, **P < 0.01, ***P < 0.001, ****P < 0.0001.

We found no reversal of the dysfunctional HBsAb response following cessation of α-TGF-β injections at week 4 post-AAV-HBV transduction, even as TGF-β levels in the depleted mice approached control levels (Fig. 1F and G). Thus, depleting TGF-β before AAV-HBV transduction resulted in persistent impairment of these responses. Since an effective antigen-specific IgG antibody response is the culmination of numerous events, both early and late after exposure to a particular antigen, we next sought to pinpoint the timeframe in which TGF-β signaling is required to promote the HBsAb response. To investigate the period during which TGF-β signaling is critical, we administered single doses of varying amounts of α-TGF-β and measured the impact on HBsAg clearance and TGF-β depletion in the serum (Fig. 3A-C). Similar to the ten-dose regimen previously used, a single 250 or 750 µg dose of α-TGF-β given 1 d prior to AAV-HBV transduction ablated active TGF-β in the periphery for at least five weeks and impaired HBsAg clearance (Fig. 3B and C). However, one 80 µg dose resulted in active TGF-β levels returning within 1 to 3 wk of AAV-HBV administration, and these mice cleared HBsAg (Fig. 3B and C). Likewise, only mice that received at least a 250 µg single dose, or the 10-dose 250 µg regimen used previously, did not generate detectable HBsAb (Fig. 3D). These results imply that TGF-β is required early post-AAV-HBV transduction, but perhaps not beyond three weeks. To confirm this, we neutralized TGF-β 3 wk after AAV-HBV administration (Fig. 3E). Mice that received the post-AAV-HBV α-TGF-β administration cleared HBsAg and generated IgG-specific HBsAb responses in the spleen (Fig. 3F and G), confirming the expendability of TGF-β beyond the early adaptive events.

### TGF-β is not required for an HBsAb response generated by VSV immunization

**Figure 4:**
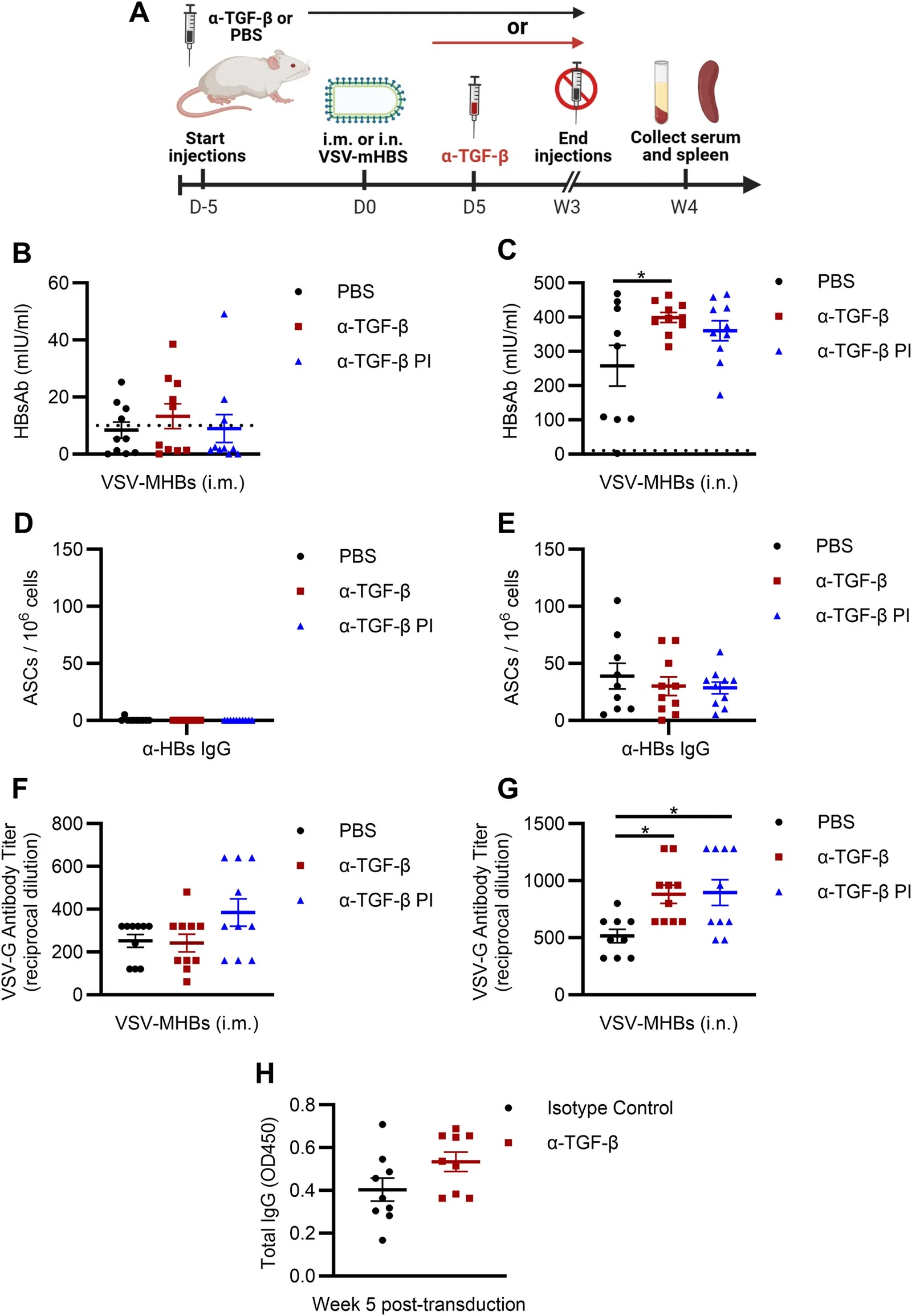
TGF-β is not required for an HBsAb response generated by VSV immunization. (A–G) BALB/c mice were given 2 × 10^6^ PFU vesicular stomatitis virus harboring middle HBsAg (VSV-MHBs) via (B, D, F) intramuscular or (C, E, G) intranasal administration at D0, and mice were euthanized four weeks post-immunization (PI), with collection of serum and spleen. Mice were administered PBS or α-TGF-β beginning 5 d prior to immunization, or α-TGF-β beginning five days after immunization, continuing twice weekly until the week 4 endpoint. (A) Experimental schematic created in BioRender. Cimino, J. (2026) https://BioRender.com/z6zg4ch. (B, C) HBsAb was measured by serum ELISA at the endpoint. Dotted lines represent the limit of detection. (D, E) Splenocytes were isolated at the endpoint, and HBs-specific IgG ASCs were quantified by ELISPOT assay. (F, G) A viral neutralization assay was performed to detect serum antibodies specific to the VSV glycoprotein (VSV-G). (H) BALB/c mice were administered α-TGF-β or isotype control beginning 5 d prior to AAV-HBV transduction, continuing twice weekly for 10 total doses, and serum was collected at week 5 post-AAV-HBV transduction for ELISA assessment of total IgG. The mean ± SEM is indicated; *N* = 9–10 mice per group. Each data point represents an individual mouse. Statistical significance was determined using 1-way ANOVA with Tukey’s or Dunnett’s test for multiple comparisons, or unpaired *t* test. *P < 0.05.

TGF-β has context-dependent roles in regulating antibody responses, including promoting IgA class switching and modulating CD4^+^ T helper cell function, and can support antigen-specific IgG production during viral infection.^38–43^ While we found that TGF-β contributes to the development of HBs-specific IgG responses in the AAV-HBV model, whether it has a general effect on the humoral response to HBsAg was unclear. To address this, naive BALB/c mice were immunized with a recombinant vesicular stomatitis virus encoding middle HBsAg (VSV-MHBs). TGF-β was inhibited either before or five days after immunization with VSV-MHBs (Fig. 4A). Neutralizing TGF-β before or after intramuscular (*i.m.*) infection did not affect HBsAb levels measured in serum (Fig. 4B). However, as this immunization did not yield a detectable splenic HBsAb response by ELISPOT (Fig. 4D), we could not assess whether HBs IgG class-switching was impaired. TGF-β depletion also did not impact the response to the viral vector itself, as there was no difference in antibodies targeting VSV glycoprotein (VSV-G), as shown by VSV neutralization assays (Fig. 4F).

To explore whether TGF-β’s impact on the HBsAb response could be dependent on the location of immune priming, we performed the same experiments using intranasal (*i.n*.) administration of VSV-MHBs. However, TGF-β depletion still did not impair antibody responses specific to HBsAg or VSV-G, and it did not impair IgG class-switching in the spleen (Fig. 4C, E, and G). In fact, TGF-β absence in the *i.n.* immunized mice slightly improved both the HBs-specific antibody response and the response to VSV-G (Fig. 4C and G). We also assessed total IgG levels in the serum of AAV-HBV-transduced TGF-β-inhibited mice and isotype control mice to determine if the absence of TGF-β might have resulted in a non-specific decrease in serum IgG. However, total IgG levels in the TGF-β-inhibited mice trended slightly higher than in control mice (Fig. 4H). Together, these results suggest that the role of TGF-β in promoting HBs-specific IgG responses may be specific to antigen expression in hepatocytes and subsequent immune priming events in the liver, rather than reflecting a broader effect on HBs-specific or total IgG antibody production.

### TGF-β influences splenic and hepatic lymphocyte responses post-AAV-HBV transduction

**Figure 5:**
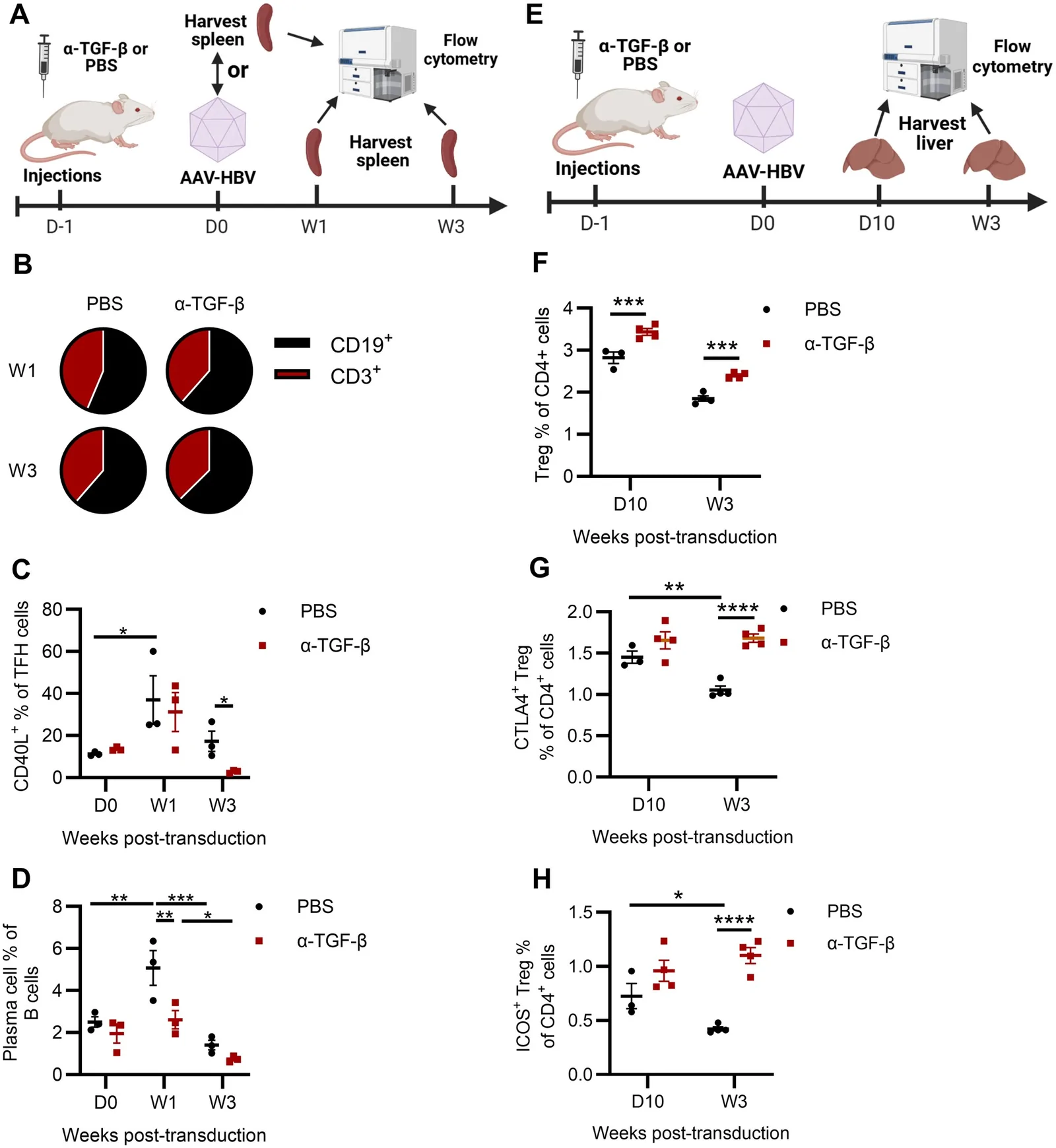
TGF-β influences splenic and hepatic lymphocyte responses post-AAV-HBV transduction. (A–D) BALB/c mice were administered one 750 µg dose of α-TGF-β or PBS 1 d prior to AAV-HBV transduction and were euthanized at either day 0 (non-transduced/naive), week 1, or week 3 post-AAV-HBV and spleen were harvested and processed for flow cytometry. (E-H) BALB/c mice were administered one 750 µg dose of α-TGF-β or PBS 1 d prior to AAV-HBV transduction and were euthanized at either day 10 or week 3 post-AAV-HBV and perfused liver were harvested and processed for flow cytometry. (A, E) Experimental schematic created in BioRender. Cimino, J. (2026) (A) https://BioRender.com/xs84mb5, (E) https://BioRender.com/euzfqev. Flow cytometry was performed to measure: (B) the proportion of T and B cells (gating on CD3^+^ or CD19^+^), (C) percentage of TFH cells that are CD40L^+^ (gating on CD3^+^ then CD4^+^ then CXCR5^+^PD1^hi^ then CD40L^+^), (D) plasma cells percentage of B cells (gating on CD19^+^ then CD138^+^GL7^-^), (F) percentage of CD4^+^ cells that are Tregs (gating on CD4^+^ then CD25^+^FoxP3^+^), (G) percentage of CD4^+^ cells that are CTLA-4^+^ Tregs (gating on CD4^+^ then CD25^+^FoxP3^+^ then CTLA-4^+^), and (H) percentage of CD4^+^ cells that are ICOS^+^ Tregs (gating on CD4^+^ then CD25^+^FoxP3^+^ then ICOS^+^). High expression was defined as the upper 50% of positive cells for the corresponding parameter. The mean ± SEM is indicated; *N* = 3–4 mice per group per timepoint. Each data point represents an individual mouse. Statistical significance was determined using 2-way ANOVA with Tukey’s test for multiple comparisons. *P < 0.05, **P < 0.01, ***P < 0.001, ****P < 0.0001.

It has previously been shown that the absence of TGF-β can distort GC dynamics in secondary lymphoid organs of mice.^44^ Because anti-HBs IgG production depends on coordinated activity between TFH cells and B cells to promote plasma cell development,^1^^,^^2^^,^^39^^,^^45^ we examined these populations in α-TGF-β-treated and control BALB/c mice following AAV-HBV transduction (Fig. 5A). To assess the impact of the α-TGF-β treatment on splenic lymphocytes in AAV-HBV-transduced mice, we analyzed CD19^+^ and CD3^+^ cells and observed no broad changes in their proportions (Fig. 5B). At three weeks post-AAV-HBV, α-TGF-β-treated mice showed a marked reduction in splenic CXCR5^+^PD-1^hi^CD40L^+^ TFH cells (Fig. 5C). Consistent with reduced TFH help, CD138^+^ plasma cells did not expand post-AAV-HBV and were significantly reduced at week 1 compared to control mice (Fig. 5D).

TGF-β promotes the peripheral induction of FoxP3^+^ Tregs and contributes to maintaining immune tolerance.^46–48^ However, TGF-β also limits conventional T cell activation, and its absence can increase IL-2 availability, promoting expansion of thymic-derived Tregs.^42^^,^^49^^,^^50^ Thus, TGF-β loss does not simply reduce Treg function but may instead alter the balance of the regulatory compartment. Therefore, we hypothesized that Tregs may be altered in the TGF-β-inhibited mice, and that such changes may contribute to the impaired HBsAb response. To assess Treg status, we focused on ICOS and CTLA-4, two markers associated with effector Treg function in chronic inflammatory settings.^51–53^ We quantified ICOS^+^ and CTLA-4^+^ Tregs in the liver to evaluate how TGF-β depletion influences this regulatory mechanism during AAV-HBV priming (Fig. 5E). Flow cytometry analyses of CD4^+^ T cells revealed a slight elevation in the fraction of hepatic Tregs in TGF-β-inhibited mice post-AAV-HBV compared with control mice (Fig. 5F). The percentage of CTLA-4^+^ Tregs was significantly increased in α-TGF-β-treated mice compared to control mice 3 wk post-AAV-HBV transduction (Fig. 5G). The proportion of ICOS^+^ Tregs was also increased in α-TGF-β-treated mice 3 wk post-AAV-HBV (Fig. 5H). Together, these data indicate that TGF-β neutralization alters B cell and CD4^+^ T cell status in the spleen and liver post-AAV-HBV transduction.

### Treg cells suppress HBsAb in mice lacking TGF-β in an IL-10-independent manner

**Figure 6:**
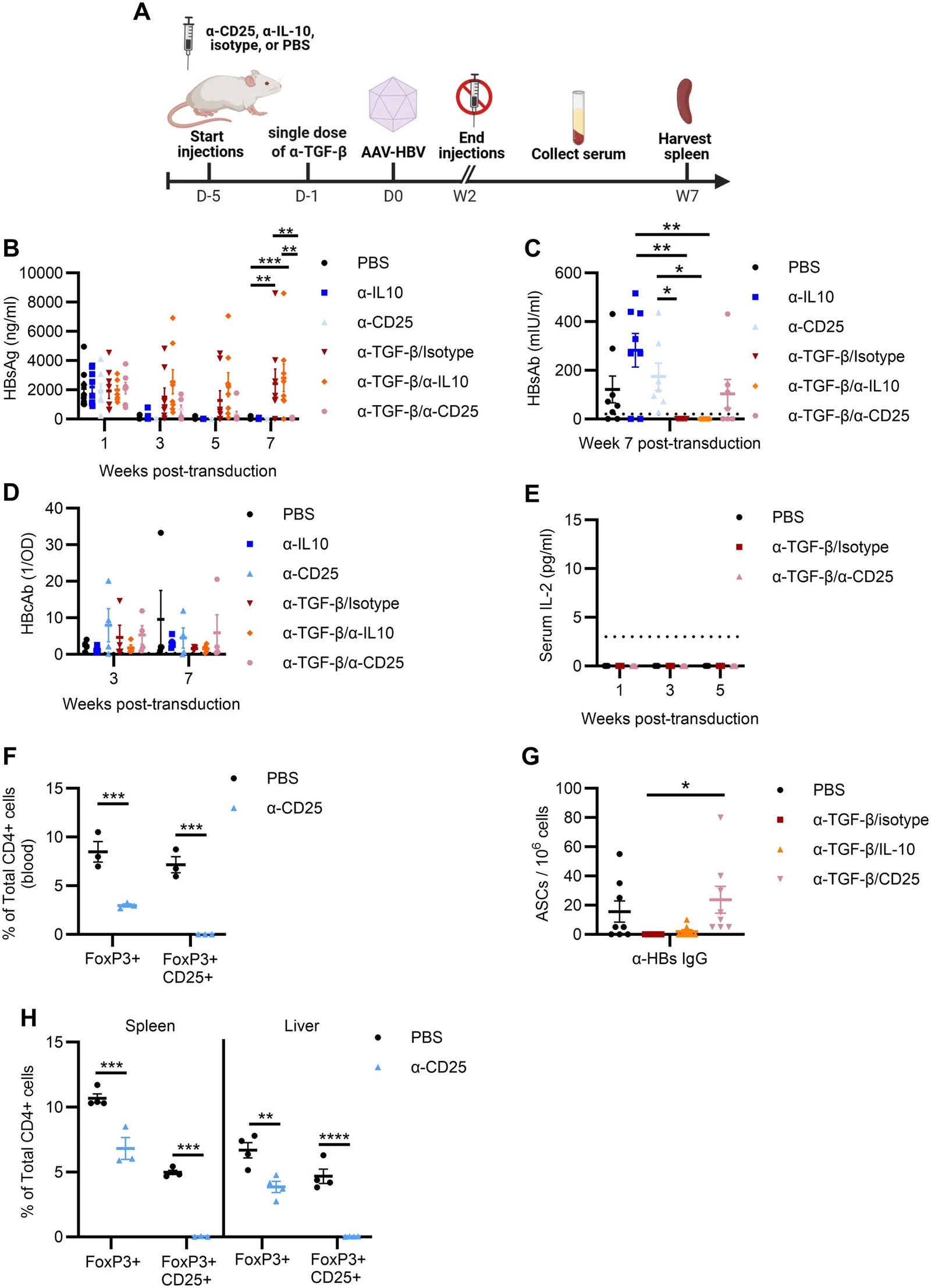
Treg cells suppress HBsAb in α-TGF-β-treated mice in an IL-10-independent manner. (A-G) BALB/c mice were administered 250 µg α-CD25, α-IL-10, isotype control, or PBS beginning 5 d prior to AAV-HBV transduction, continuing twice weekly for 4 total doses. Mice receiving α-TGF-β received one single 750 µg dose 1 d before AAV-HBV, and serum and spleen were collected. (A) Experimental schematic created in BioRender. Cimino, J. (2026) https://BioRender.com/ztk8rc5. ELISAs were performed to measure serum (B) HBsAg, (C) HBsAb, (D) HBcAb, and (E) IL-2. (F) Blood was collected from PBS or α-CD25 mice at week 1 post-AAV-HBV, or (H) spleen and liver of PBS or α-CD25 treated non-transduced mice were harvested, and Treg cells were quantified by flow cytometry. The percentages of total CD4^+^ cells that are FoxP3^+^ or FoxP3^+^/CD25^+^ are shown (gating on CD4^+^ then FoxP3^+^ or FoxP3^+^/CD25^+^). (G) Splenocytes were isolated at the week 7 endpoint, and HBs-specific IgG ASCs were quantified by ELISPOT analysis. The mean ± SEM is indicated; *N* = 6–8 mice per group, except for IL-2 ELISA, where pooled serum was used for a total of *N* = 4 per group (8 mice per group, pooled in pairs), and flow cytometry where *N* = 3–4 mice per group. Except for panel (E), each data point represents an individual mouse. Statistical significance was determined using one-way or two-way ANOVA with Kruskal–Wallis, Tukey’s, or Šidák’s test for multiple comparisons. *P < 0.05, **P < 0.01, ***P < 0.001, ****P < 0.0001.

Based on these findings, we next explored whether Tregs mediate suppression of the HBsAb response in the absence of TGF-β. Thus, we depleted Tregs (α-CD25) or neutralized IL-10 in α-TGF-β-treated BALB/c mice (Fig. 6A). As observed previously (Fig. 2), neither intervention alone altered HBsAg clearance nor HBsAb production (Fig. 6B and C). However, combined Treg and TGF-β depletion, but not IL-10 and TGF-β depletion, restored both HBsAg clearance and robust HBs-specific antibody responses (Fig. 6B, C, G). Related to the previous experiments assessing the specificity of the TGF-β effect on the antibody response (Fig. 4), there was no difference between TGF-β-treated mice and the other experimental groups in anti-HBc antibody production (Fig. 6D).

One of the major IL-10-independent mechanisms Tregs can use to suppress adaptive responses is by sequestering IL-2 using its high-affinity IL-2Rα (CD25).^54^^,^^55^ Since IL-2 is a critical cytokine that promotes maturation of CD4^+^ T cells,^54^^,^^55^ we determined whether CD25 depletion in TGF-β-inhibited mice resulted in a significant increase in IL-2 levels that could correlate with the improved HBs humoral response. However, there was no detectable increase in serum IL-2 in these mice (Fig. 6E). Efficacy of the α-CD25 treatment to deplete Treg cells or block CD25 was confirmed in the blood, spleen, and liver by flow cytometry (Fig. 6F and H). Together, these findings highlight that the suppression of the HBsAb response in the absence of TGF-β is at least partially Treg-dependent but is independent of IL-10.

### Compensatory suppression in the absence of TGF-β is mediated in part by PD-1

**Figure 7:**
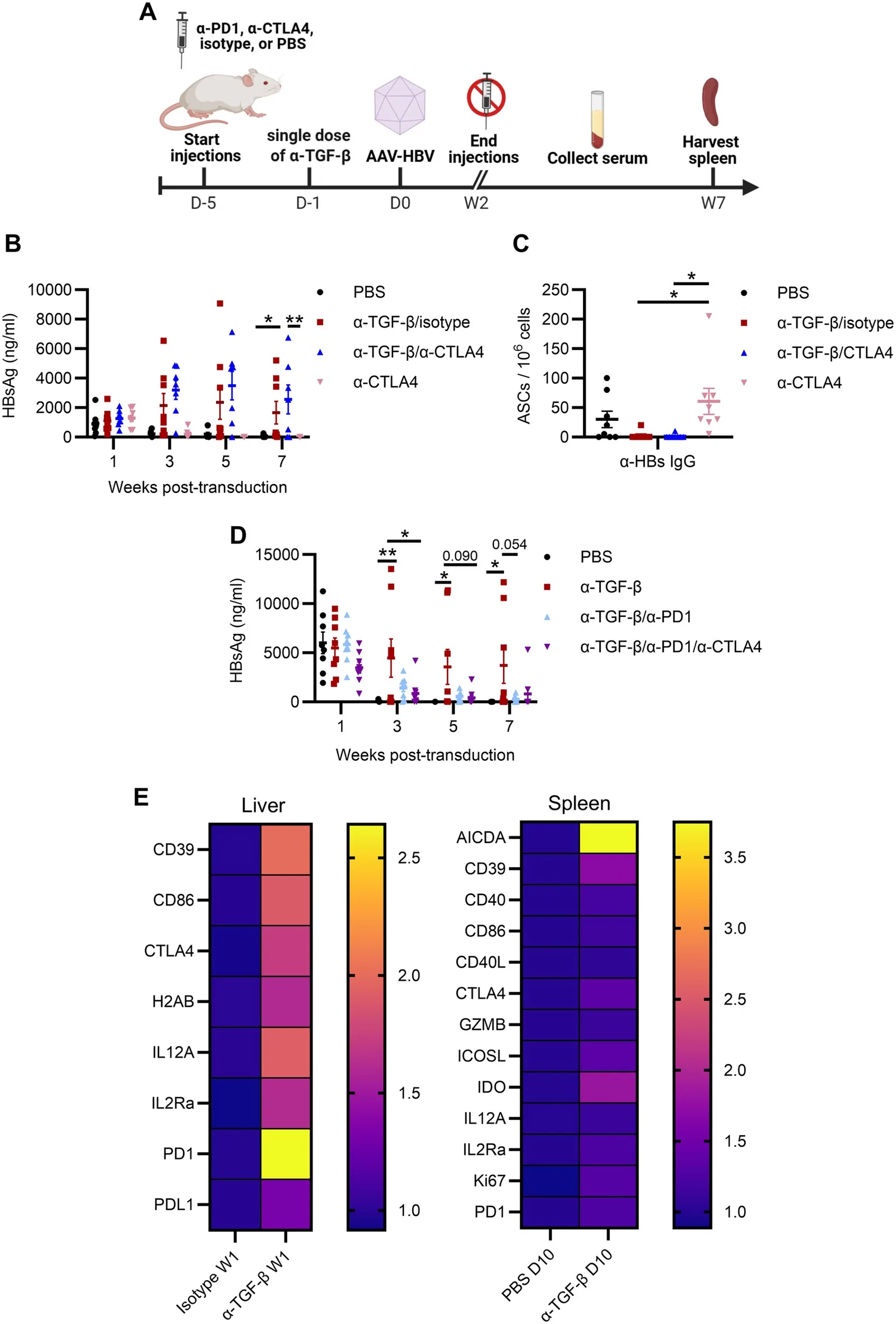
PD-1, but not CTLA-4 alone, suppresses the humoral response to HBsAg in the absence of TGF-β. (A–D) BALB/c mice were administered 250 µg of α-CTLA-4, 250 µg of α-PD-1, 250 µg of both, isotype, or PBS. Mice receiving α-TGF-β received one single 750 µg dose 1 d before AAV-HBV, and serum and spleen were collected. (A) Experimental schematic created in BioRender. Cimino, J. (2026) https://BioRender.com/niuwmmh. (B, D) Serum ELISAs to assess HBsAg over time. (C) Splenocytes were isolated at the week 7 endpoint, and HBs-specific IgG ASCs were quantified by ELISPOT analysis. (E) Liver (left) or spleen (right) were harvested from AAV-HBV mice at week 1 or day 10 post-transduction. RT-qPCR was performed to quantify RNA expression of various targets, and data are shown as heatmaps visualizing the ratio of target RNA to GAPDH endogenous control. Data are normalized to mean expression in isotype (left) or PBS (right) control mice. The left and right panels are from separate experiments. The mean ± SEM is indicated; *N* = 8 mice per group, except qPCR data, which represents *N* = 3 mice per group per timepoint. Each data point in (B), (C), and (D) represents an individual mouse. Statistical significance was determined using 2-way ANOVA with Tukey’s test for multiple comparisons. *P < 0.05, **P < 0.01, ***P < 0.001, ****P < 0.0001.

Because CTLA-4 expression was increased on hepatic Tregs in α-TGF-β–treated mice (Fig. 5), we also tested whether CTLA-4 signaling contributed to the HBsAb suppression seen in TGF-β-inhibited mice (Fig. 7A). Like IL-10, and in contrast to Treg depletion, CTLA-4 blockade did not restore HBsAg clearance or HBs-specific antibody responses in α-TGF-β-treated mice (Fig. 7B and C), indicating that the regulatory mechanism limiting HBsAb responses in the absence of TGF-β is also CTLA-4-independent. PD-1 is often therapeutically inhibited in combination with CTLA-4 in other disease models, including cancer, as the dual treatment synergizes to help overcome redundant and non-redundant suppressive capabilities.^56^^,^^57^ Therefore, we also blocked PD-1 or PD-1 and CTLA-4 in mice lacking TGF-β (Fig. 7A) and found that PD-1 blockade alone was sufficient to promote clearance in TGF-β-inhibited mice (Fig. 7D). This finding suggests PD-1 is a compensatory driver of the humoral suppression observed in mice lacking TGF-β.

Immune regulation can also be achieved through altered metabolism of immune cells, and factors such as the ectonucleotidase CD39 and the enzyme indoleamine 2,3-dioxygenase 1 (IDO1) have potent inhibitory effects on B and T cells that can disrupt humoral immunity to viruses and potentiate chronic disease.^58–61^ Furthermore, CD39 and IDO1 are well-documented mechanisms by which Tregs can limit effector T cells and impact adaptive immunity.^62–64^ To determine if metabolic suppression could be another compensatory mechanism indicated here, we quantified RNA expression in the liver and spleen early post-AAV-HBV (Fig. 7E). CD39 was upregulated in α-TGF-β-treated mice in the liver and spleen early post-AAV-HBV, while IDO1 was upregulated in the spleen. These data also revealed expression of genes associated with other possible mechanisms of suppression, including elevated IL-12A (a component of IL-35) and PD-1 in the livers of TGF-β-inhibited mice (Fig. 7E), as well as splenic elevation of AICDA, which has been associated with GC dysfunction in other chronic diseases like cancer.^65^^,^^66^

## Discussion

TGF-β, IL-10, and Tregs are critical immune regulators that can impact the inflammatory balance during the innate and adaptive responses to foreign and self-antigens. Numerous studies have shown that TGF-β can not only regulate various immune cells, but in particular, can promote adaptive responses, including class-switched antigen-specific antibody responses.^38–40^^,^^67–72^ Here, TGF-β promoted the antigen-specific humoral response to HBsAg, underscoring its role in providing adaptive immune support to a viral infection. With TGF-β’s absence before and during the first 3 wk of HBsAg exposure, there was irreversible impairment of the HBsAb response. This effect may be specific to the early HBsAg immune priming that occurs in the liver after AAV-HBV transduction, as the humoral response to i.m. and i.n. immunization with VSV-MHBs was not impaired by TGF-β depletion and there was no impairment of the anti-HBc response. However, further studies using AAV harboring secretion-deficient HBsAg or other T-cell-dependent antigens could help identify the specificity of this effect.

One nuance in studying the dynamics of HBs-specific responses concerns the formation of immune complexes. Depending on the stage of infection, humans can have very high levels of HBsAg in the blood.^73–75^ We previously found that high HBsAg loads can contribute to adaptive immune dysfunction in AAV-HBV mice, and prolonged exposure to large quantities of HBsAg contributes to the immune tolerance observed in CHB patients.^76–79^ This high antigen load means that an antibody surplus is needed to bind all free HBsAg. Thus, a majority of HBsAb may be bound to HBsAg, and as a result, it can take many weeks after AAV-HBV administration before the concentration of HBsAb exceeds the amount of HBsAg produced by transduced hepatocytes. HBsAb bound in complex with HBsAg is not detected by the ELISA used here, resulting in low to undetectable HBsAb early post-AAV-HBV, which rises over time in mice that have generated HBs-specific lymphocytes. However, measuring HBs-specific IgG secretion in whole splenocytes enabled the detection and assessment of class-switched HBsAb responses post-AAV-HBV.

Unlike natural HBV infection, AAV vectors are recognized by multiple innate sensors, including TLR9, and can activate type 1 interferon responses.^80^ This may be considered when translating findings from this model to humans; nevertheless, these innate signals are not sufficient to induce effective humoral or cytotoxic responses to HBV in many mouse strains, including C57BL/6. BALB/c and C57BL/6 mice differ in their ability to efficiently generate an HBsAb response that effectively controls HBsAg, with only BALB/c mice being able to generate HBs-specific IgG antibodies, while neither naturally develops a cytotoxic CD8 T cell response that clears infected hepatocytes.^21–24^ Furthermore, there is no reinfection of hepatocytes after AAV-HBV transduction initially delivers the HBV genome, meaning the model is not a perfect replica of human infection. Despite these limitations, the AAV-HBV model offers distinct advantages over other models, enabling temporal studies that exploit known strain-specific differences in the response to HBV to characterize and define adaptive immune factors and mechanisms, including early regulatory dynamics following hepatic HBV antigen exposure.^18^^,^^21^ As shown here, these strains also differ in how TGF-β influences their humoral response. TGF-β has no impact on the HBsAb response in C57BL/6 mice, yet it is necessary in BALB/c mice early following exposure to HBV. BALB/c mice tend to have higher basal levels of TGF-β than C57BL/6 mice,^81^^,^^82^ and since TGF-β signaling was critical to the HBsAb response in BALB/c mice, it is possible that enhancing TGF-β signaling in C57BL/6 mice could promote an HBsAb response. However, due to its pro-fibrotic and immunosuppressive properties, therapeutic activation of TGF-β can have various side effects and confound immune analyses, so this possibility was not addressed here.^81^^,^^83–86^

The finding that serum HBeAg was decreased, although not cleared, in TGF-β-inhibited mice was not due to decreased viral gene expression, as evidenced by similar HBV RNA levels in the liver at both early and late time points post-AAV-HBV. Similar to control mice, there was no detectable HBeAb in the serum of mice lacking TGF-β. Like the previously mentioned inability of the ELISA to detect HBsAg in complex, HBeAb could be present in higher amounts than control mice but still not high enough for the free antibody to be detected by the assay. However, the fact that HBeAg was still not cleared from the periphery nearly 3 months post-AAV-HBV indicates that this possibility is less likely. Alternatively, the decreased HBe antigenemia may be a result of impaired intrahepatic viral protein translation or antigen secretion from hepatocytes or improved non-antibody-mediated clearance. Our gene expression data showing enhanced expression of CD86, H2-AB, and IL-12A in the livers of TGF-β-inhibited mice support that certain aspects of intrahepatic immune activation including antigen presentation may be bolstered, which could result in elevated phagocytic antigen clearance independent of humoral neutralization. Regardless, the enhanced HBeAg clearance did not correlate with a CD8^+^ T cell response necessary to eliminate infected hepatocytes, as evidenced by the lack of HBV-specific T cells in the spleen and stable HBV gene expression in the liver. Studies incorporating TGF-β depletion and assessing antigen-specific splenocytes with in vitro and ex vivo techniques or quantifying early HBe-specific IgM responses could aim to further explore the impact of TGF-β on HBeAg secretion and HBeAb production.

The TGF-β dependency being restricted to the first 3 wk following AAV-HBV transduction suggests that its main function is in shaping early priming or adaptive differentiation rather than maintaining effector function long-term. This window coincides with initial CD4^+^ T cell priming, TFH commitment, and GC establishment, which may parallel the timing of early adaptive events that lead to HBsAg seroconversion in humans.^1^^,^^2^^,^^38^^,^^87^ Expression analyses also revealed early alterations in genes that could influence hepatic immune priming in response to AAV-HBV. However, whether TGF-β regulates the humoral response directly or indirectly, and on B cells or T cells, remains uncertain. Failure of IL-21R blockade to impair HBsAb generation signifies that other pathways supporting effective B cell maturation and differentiation may be sufficient. Studies incorporating B cell or T cell-specific TGF-βR knockout mice would help further distinguish the mechanism.^43^^,^^88^^,^^89^ Exploring the possibility that TGF-β stabilizes antigen presentation or regulates the function of critical APCs, such as follicular dendritic cells, also warrants future consideration.

We initially found no impact of Tregs or IL-10 on the HBsAb response in BALB/c mice. There are several examples of immune compensation and redundant pathways involving TGF-β and IL-10 with and without the help of Tregs, and TGF-β’s documented role in CD4^+^ T cell development and maintenance makes it evident that Tregs could be rewired in its absence.^77^^,^^90^^,^^91^ For example, TGF-β can have compensatory upregulation in the absence of IL-10 signaling, and it was also found that TGF-β signaling is not required to retain Treg suppression in a colitis model.^92–94^ Here, we found that Tregs appear to take on a role as humoral suppressors in the absence of TGF-β in an IL-10-independent manner, as unlike Treg depletion in TGF-β-inhibited mice, neutralization of IL-10 did not restore HBsAb responses.

Several major mechanisms have been established by which Tregs can suppress adaptive immune responses that do not rely on IL-10 signaling, including restricting effector T cells by sequestration of IL-2.^95^ However, one of the most powerful mechanisms is mediated cell-to-cell by the immune checkpoint protein CTLA-4.^96^^,^^97^ Not only was the percentage of CTLA-4^+^ hepatic Tregs elevated in TGF-β-inhibited mice, but so were Tregs expressing the costimulatory receptor ICOS, indicating potential mechanisms by which these cells could be inducing tolerance to HBsAg. Nevertheless, CTLA-4 blockade did not promote HBsAg clearance in TGF-β-inhibited mice. It is possible that administering the CTLA-4 blockade prior to AAV-HBV transduction may have triggered early immune compensation that stunted the priming or development of HBs-specific responses, as several studies have shown that CTLA-4’s impact on effector cells can yield distinct, context-dependent outcomes.^98–100^

However, there are several alternative mechanisms employed by Tregs that could also suppress HBsAb in the absence of both TGF-β and CTLA-4. We saw no detectable increase in serum IL-2 but observed elevated expression of genes associated with metabolic regulatory pathways including CD39 and IDO-1, indicating that when TGF-β is absent there could be enhanced restraint on ATP availability for adaptive immune cells. While we observed no increase in hepatic granzyme B gene expression in the liver of TGF-β-inhibited mice one week post-AAV-HBV, there was elevated hepatic expression of IL-12A. Studies on a cellular level would be needed to confirm this, but these findings suggest that granzyme B production by B regulatory cells may not be involved, yet IL-12A, one component of the known immune suppressor IL-35, may be a possible driver of compensation.^101–104^

It remained feasible that other checkpoint proteins such as PD-1 are compensating for the lack of CTLA-4. Interestingly, PD-1 blockade restored HBsAg clearance in TGF-β-inhibited mice. It is possible that PD-1, a prominent controller of TFH and GC B cells,^105^^,^^106^ or its ligand PD-L1 are upregulated in the absence of TGF-β, resulting in impaired humoral responses. Ultimately, this finding implicates PD-1 as at least one of the drivers of the humoral suppression observed in the absence of TGF-β, but as many other immune cells including B cells can express PD-1,^107^ further studies assessing PD-1 expression on Tregs or utilizing PD-1 deficient Tregs would be needed to conclude if Tregs are mediating their suppression through the PD-1 axis.

TGF-β, IL-10, and Tregs have long been associated with suppressive functions in chronic diseases, including cancer, viral infections, and autoimmunity. This work shows that during early HBV infection, there may be direct or indirect roles for these factors that are less documented. Exploring TGF-β polymorphisms in human patients may be warranted to determine if a correlation exists that could help identify patients at risk of developing CHB. While activating TGF-β may be therapeutically challenging, it is worth exploring if manipulating other factors upstream or downstream of TGF-β signaling in early HBV infection could promote acute clearance and prevent tolerance to HBsAg and subsequent chronic infection. New insights into the early immune regulatory interactions that impact humoral immunity can help guide therapies aimed to bolster or generate HBsAb responses, with clinical relevance for vaccine non-responders or CHB patients to stimulate HBsAg seroconversion. Together, these data further elucidate the feedback and interplay between major immune regulatory factors and highlight potential interactions and mechanisms that may contribute to the progression towards chronic manifestation of HBV.

## Data Availability

The data supporting the findings of this study are available from the corresponding author upon reasonable request.
